# Identifying new players of gynoecium development using tissue-specific transcriptome data of Arabidopsis

**DOI:** 10.1007/s00425-025-04784-0

**Published:** 2025-07-31

**Authors:** Eliana Arias-Pérez, Valentín Luna-García, Judith J. Bernal-Gallardo, Stefan de Folter

**Affiliations:** Centro de Investigación y de Estudios Avanzados del Instituto Politécnico Nacional (Cinvestav), Unidad de Genómica Avanzada (UGA-Langebio), 36824 Irapuato, Mexico

**Keywords:** Fertilization, Fruit, Gynoecium, Pollen tube, Seeds, Septum, Transcriptome, Transmitting tract

## Abstract

**Main conclusion:**

We identified new players of male and female reproductive development using tissue-specific gynoecium transcriptome data of Arabidopsis.

**Abstract:**

Reproductive success in angiosperms depends on the correct development of the male and female organs. Pollen grains will land and germinate on the stigma, followed by the growth of pollen tubes that pass through the transmitting tract system of the gynoecium to reach the ovules. After the fertilization process occurred, seed and fruit development initiates. Genetic networks direct these biological processes needed for flower and fruit development to occur. Although many genes have been identified, still genes are to be discovered also to be involved in these networks. The availability of transcriptomic data from specific tissues of the gynoecium in Arabidopsis allowed us to select genes for functional analysis. As a result, from the analysis of the mutant plants we found that the genes *CLE19*, *TBL36*, *ATHB5*, *CYCP4;1*, *AT3G06035,* and *AT1G15760* affect fertility in Arabidopsis. The mutant plants showed gynoecia with aborted ovules and short fruits with a lower number of seeds compared to wild type. Furthermore, pollen development and pollen tube growth were affected in most of the mutants. These results help us know and understand the genes that contribute to flower development in Arabidopsis.

**Supplementary Information:**

The online version contains supplementary material available at 10.1007/s00425-025-04784-0.

## Introduction

The development of flowers and that of fruits are adaptive advantages of sexual reproduction in angiosperms (Armbruster 2014). In Arabidopsis, the floral architecture is composed of organs that are concentrically distributed in four whorls, from the outside to the inside: sepals, petals, stamens (male organ), and carpels (female organs). The carpel corresponds to the female part of the flower and can be formed by one or more carpels. In Arabidopsis, two congenitally fused carpels form the gynoecium also called pistil. At the margins where the carpels fuse, a meristematic tissue called carpel margin meristem (CMM) is formed, which gives rise to the placenta, ovules, septum (SEP), transmitting tract, style, and stigma, all of which tissues are important for the reproductive success of the plant (Roeder and Yanofsky 2006; Alvarez-Buylla et al. 2010; Ferrándiz et al. 2010; Reyes-Olalde et al. 2013; Simonini and Østergaard 2019). In Arabidopsis, the septum is the specialized central tissue that originates from the fusion of the margins of the carpels within which the transmitting tract is formed. In the reproductive process, pollen tubes enter the transmitting tract from the style and travel basally to reach and fertilize the ovules (Crawford and Yanofsky 2008; Reyes-Olalde et al. 2013; Reyes-Olalde and de Folter 2019; Pereira et al. 2021). In general, after fertilization of the ovules, the gynoecium transforms into a fruit that protects the developing seeds until they are dispersed into the environment (Ballester and Ferrándiz 2017).

Elucidating the genes and genetic networks that contribute to gynoecium and fruit development has been a central theme in plant biology (Zúñiga-Mayo et al. 2019; Herrera-Ubaldo and de Folter 2022; Ramos-Pulido and de Folter 2023; Ding et al. 2024). Advances in the identification of key regulators involved in gynoecium patterning have been well studied in *Arabidopsis thaliana*. These networks integrate different families of transcription factors, hormones, peptides, and proteins, among others, which together define carpel identity and contribute to gynoecium development and fruit formation (Chávez Montes et al. 2015; Herrera-Ubaldo and de Folter 2022). Examples of genes that are involved in the development of specific tissues derived from the CMM are *SPATULA* (*SPT*), *CUP-SHAPED COTYLEDON* (*CUC1,2*), *HECATE* (*HEC1-3*), *LEUNIG* (*LEU*), *HALF FILLED*/*CESTA* (*HAF*/*CES*), and *NO TRANSMITTING TRACT* (*NTT*), among others (Liu and Meyerowitz 1995; Ishida et al. 2000; Heisler et al. 2001; Gremski et al. 2007; Crawford et al. 2007; Crawford and Yanofsky 2011; Kamiuchi et al. 2014).

**Fig. 1:**
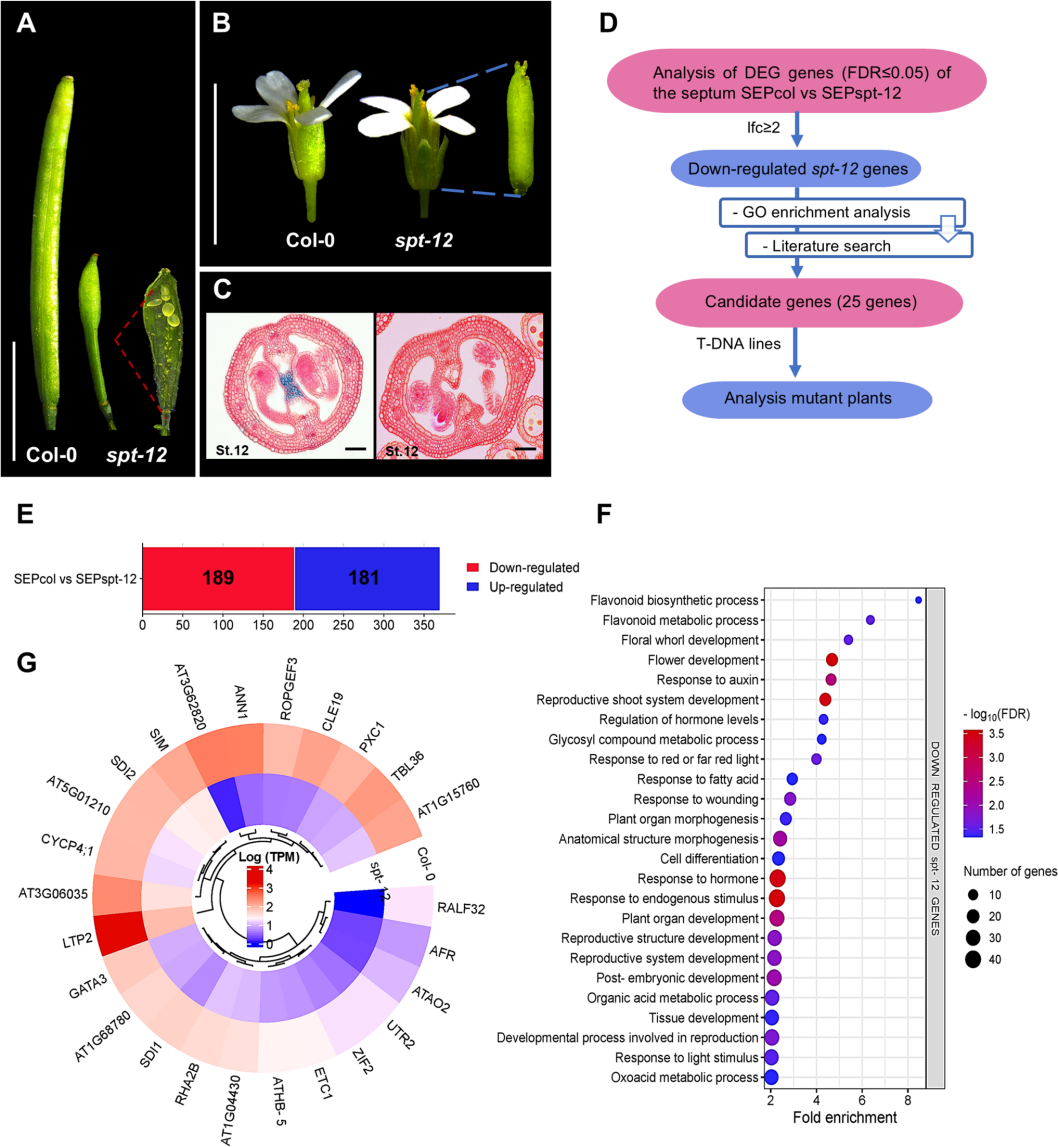
Identification of differentially expressed genes (DEGs) in septum tissue based on RNA-seq data from wild-type (Col-0) Arabidopsis plants versus *spatula* mutants (*spt-12*). **A**–**C** Flower and fruit phenotypes in wild-type (Col-0) and *spt-12* mutant plants. **D** An overview of the gene selection process. **E** Number of up- and down-regulated genes (FDR ≤ 0.05, Log2FC ≥ 2). **F** Biological process enriched terms for the down-regulated genes. **G** Expression levels of the 25 selected genes (Log_2_ TPM). Scale bars = 5 mm (**A**, **B**), 50 µm (**C**)

In Arabidopsis, the loss of function of the transcription factor *SPT* has been widely studied. The *SPT* gene is required for the correct development and postgenital fusion of the septum. In the *spt* mutants, septum formation is disrupted, resulting in a failure of carpel fusion. Moreover, the transmitting tract is absent. In addition, the development of the stigmatic papillae is reduced and there is a defect in the fusion of the two carpels in the apical region. These defects are also evident in developing fruits or siliques. Despite this, fertilization and seed development occur, although at a drastically reduced proportion. Fruits of the *spt* mutant are shorter than wild-type fruits and wider in the medial plane, showing a spatula-like appearance (Fig. 1) (Alvarez and Smyth 1999, 2002; Heisler et al. 2001; Groszmann et al. 2010).

Currently, transcriptomic data of specific tissues of the gynoecium are available: presumptive carpel wall (PC), carpel margin meristem (CMM), septum (SEP), and valves (VV), both for the wild-type Col-0 and the *spt-12* mutant plants (Luna-García et al. 2024; Luna-García et al., data not shown). With the availability of this transcriptomic data, we can study and identify possible regulators for the development of inner tissues of the gynoecium such as the septum. Therefore, in the present work, the selection of candidate genes from these transcriptomic data was carried out focusing on the septum to perform the functional analysis, with the aim of identifying regulators that orchestrate the development of the gynoecium and fruit in Arabidopsis. As a result, we found reduced fertility phenotypes in mutant plants for six genes.

## Materials and methods

### Transcriptomic data analysis and gene selection

For the selection of candidate genes involved in medial tissue development, the septum samples from the high-resolution gene expression maps of the medial and lateral domain of Arabidopsis gynoecia from wild type and the *spatula* (*spt-12*) mutant were used (Fig. 1), which we previously generated (Luna-García et al. 2024; Luna-García et al., data not shown). The data are available in the European Nucleotide Archive (ENA) under accession numbers PRJEB65130 (Col-0) and PRJEB79454 (*spt-12*), and in the eFP browser (Winter et al. 2007): https://bar.utoronto.ca/efp/cgi-bin/efpWeb.cgi?dataSource=Gynoecium. We used the differentially expressed genes (DEGs) from the septum (SEP) samples between the wild type and the mutant (SEP, stage 10 of gynoecium development); the list of DEGs is the result of the analysis in EdgeR comparing the gene expression of SEPcol versus SEPspt-12 (FDR ≤ 0.05), and applying a threshold of a log_2_ fold change (Log2FC) ≥ 2 (Table S1). We focused on the down-regulated genes in *spt-12* (Fig. 1E). A GO enrichment analysis of the biological processes was performed using the online tool PHANTER 17.0 (https://www.pantherdb.org/), with applying the Fisher’s Exact statistical test and FDR ≤ 0.05 (Fig. 1F, Table S2). The results were graphed using the ggplot2 package in R (Fig. 1F) (R Core Team 2022). Information on the first 100 genes was also searched on the TAIR website (https://www.arabidopsis.org/) to find information about the genes and to find out if they had already been characterized. Finally, 25 candidate genes were selected to study in this work (Fig. 1G).

### Plant material and growth conditions

*Arabidopsis thaliana* plants used in this study were wild-type Columbia (Col-0) plants, *spt-12* mutant (Ichihashi et al. 2010), and mutant lines for all 25 genes (Table S3) were obtained from the Arabidopsis Biological Resource Center (ABRC, https://abrc.osu.edu/). All were germinated in soil (3:1:1, peat: perlite: vermiculite) in a growth chamber under long-day conditions (16 h light/8 h dark, 22 °C) for 2 weeks. Seedlings were then moved to greenhouse conditions (22–27 °C, natural light).

### Phenotypic analysis

The phenotypic analysis consisted of first a general screening of the mutant lines for the 25 genes [41T-DNA lines (for various genes more alleles were available); Table S3], which consisted of the observation of the development of flowers, gynoecia, and fruits. Subsequently, we focused on the lines that presented a phenotype by growing subsequent generations, and which were genotyped (Fig. S1, Table S4). Detailed analyses consisted in determining the length of the gynoecium and fruit, and counting the number of ovules and seeds in wild-type Col-0 and mutant plants. The measurements of gynoecia and fruits were made in the ImageJ program (https://imagej.net/ij/). Statistical tests of the measurements were analyzed by ANOVA followed by a post hoc Tukey test for the inference of a significant statistical difference with an *α* = 0.05 using the Rstudio program (R Core Team 2022).

### In silico analysis of the gene expression profile

To visualize the expression profile of the genes of which their mutants showed interesting phenotypes, a heatmap was made using the transcriptomic data generated by Luna-García et al. (2024) and Luna-García et al. (data not shown). The expression data in TPM (transcripts per million) were transformed to a logarithmic scale (Log_2_) and subsequently a heatmap was performed with the pheatmap package in R (R Core Team 2022).

The expression of the genes in specific tissues of the gynoecium: presumptive PC, CMM, SEP and valves (VV) was also visualized using the eFP Browser tool (https://bar.utoronto.ca/efp_arabidopsis/cgi-bin/efpWeb.cgi?dataSource=Gynoecium).

### Histological analysis and staining

Pollen viability was analyzed using Peterson staining (Peterson et al. 2010). Flowers at stages 12 and 13 were collected and fixed in Carnoy’s fixative (alcohol:chloroform:acetic acid, 6:3:1, by vol.) for 2 h. Anthers were then dissected using a Leica EZ4 D stereomicroscope (Leica, Wetzlar, Germany) and placed on a slide to which two to four drops of the staining solution was added. The slide was then slowly heated over an alcohol burner in a fume hood for approximately ~ 30 s and a coverslip was placed over the sample. Photographs were taken with a DM6000B microscope (Leica).

Pollen tube growth was assessed using aniline blue staining as previously described (Jiang et al. 2005). Wild-type and mutant flowers were emasculated just before pollination (late stage 12), and after 24 h they were manually pollinated (8 to 10 gynoecia). The next day, gynoecia were collected and fixed in ethanol:acetic acid (3:1, v/v) for 2 h at room temperature. Subsequently, three washes were performed with sterile distilled water and the samples were treated with 8 M NaOH overnight. Afterward, gynoecia were washed three times with distilled water and stained with aniline blue solution (0.1% aniline blue (w/v) in 150 mM K_2_HPO_4_ buffer, pH 11) for 5 h in the dark. Finally, gynoecia were observed and photographed with a DM6000B fluorescence microscope under UV light (Leica).

To analyze transverse gynoecia, inflorescences of the wild type and mutants were collected and fixed in FAE (3.7% formaldehyde, 5% glacial acetic acid, and 50% ethanol), with vacuum (15 min, 4 °C), and then incubated for 60 min at room temperature. The FAE was then removed, and the material was rinsed with 70% ethanol and incubated overnight at 4 °C in 70% ethanol, followed by dehydration in a series of ethanol concentrations (70, 85, 95, and 100%) for 60 min each and embedded in Technovit 7100 (Heraeus Kulzer) according to the manufacturer’s instructions. Blocks were sectioned (10–12 µm) on a rotary microtome (Leica). To visualize the transmitting tract, tissue sections were stained with a 0.5% alcian blue solution (pH 3.1; Sigma-Aldrich) and counterstained with a 0.5% neutral red solution (Sigma-Aldrich) as previously described (Zúñiga-Mayo et al. 2012). For the *cycp4;1* mutant, the number of cells in the septum and replum was counted, and the student’s t-test statistical analysis was performed.

### RNA extraction and RT-PCR

Total RNA was extracted from inflorescences using TRIzol (Invitrogen). After treatment with DNase I (Invitrogen), cDNA was prepared using M-MLV reverse transcriptase according to the manufacturer’s instructions (Invitrogen). Semi-quantitative RT-PCR was performed using a thermal cycler (Applied Biosystems, Thermo Fisher Scientific). Three biological replicates and two technical replicates were used for each assay. The *ACTIN2* gene was used as a control (Fig. S2). The primer sequences used and PCR conditions are shown in Table S4.

## Results

### Transcriptomic data analysis: gene selection

With the aim to find new regulators of medial tissue development of the Arabidopsis gynoecium, we used transcriptome data from Laser-Assisted Microdissection (LAM) combined with RNA-seq from wild type and the *spt-12* mutant, a mutant affected in medial tissue development (Fig. 1A–C; Luna-García et al. 2024; Luna-García et al., data not shown; see Materials and methods). The selection process of the candidate genes for this study consisted of comparing the transcriptome data from cells of the septum (medial tissue) from gynoecia of wild-type *A. thaliana* Col-0 and the *spt-12* mutant plants (Fig. 1D). Differentially Expressed Genes (DEGs) were called using EdgeR by contrasting septum tissue (SEPcol vs SEPspt-12; FDR ≤ 0.05, Log2FC ≥ 1; Table S1). A total of 1151 DEGs were found in this tissue, and to reduce the list with candidates, we applied a cut-off of Log2FC ≥ 2, and obtained a total of 370 DEGs, with 181 up-regulated and 189 down-regulated genes (Fig. 1E, Table S1). We decided to focus on the down-regulated genes in the *spt-12* mutant, with the idea that these genes might be involved in medial tissue development. Analyzing the list of these 189 DEGs, we observed many genes that have already been reported to be involved in gynoecium development such as *SPATULA (SPT)* (Alvarez and Smyth 1999; Heisler et al. 2001), *CUC1* (Ishida et al. 2000; Cucinotta et al. 2014; Gonçalves et al. 2015), *NGATHA2* (*NGA2*) and *NGA3* (Alvarez et al. 2009; Trigueros et al. 2009; Fourquin and Ferrándiz 2014), *TAA1* (Nole-Wilson et al. 2010; Tan et al. 2023), *PIN3* (Reyes-Olalde et al. 2017; Müller et al. 2017), *WOX6* (Park et al. 2005; Xiang et al. 2011), *YUCCA4* (*YUC4*) and *YUC7* (Cheng et al. 2006, 2007; Yamaguchi et al. 2018), *REM20* (*VDD*) (Matias-Hernandez et al. 2010; Mendes et al. 2016), and *BEE1* (Crawford and Yanofsky 2011; Di Marzo et al. 2020).

Furthermore, we verified the biological processes that are enriched in this set of DEGs, so a Gene Ontology (GO) enrichment analysis was performed, where we found enriched categories related to cell differentiation processes, development and morphogenesis of floral tissues and organs, regulation and response to hormones, response to red and far-red light, response to fatty acids, and post-embryogenic development, among others (Fig. 1F, Table S2). It is important to mention that *SPT* has been shown to be involved in some of these biological processes (Foreman et al. 2011; Reymond et al. 2012; Liu et al. 2017; Reyes-Olalde et al. 2017).

We also searched for information in the literature for the top 100 down-regulated DEGs, and to select candidate genes to study, we decided to focus on genes that had not been characterized or those with few or no reports, in addition to the availability of T-DNA lines. Finally, we selected 25 genes that we considered interesting to study their possible function in flower (gynoecium) and fruit development. Based on the transcriptome data, all these genes had lower expression levels in the septum of the *spt-12* mutant compared to the wild type (Col-0) (Fig. 1G, Table S3).

### Gene information and expression profile analysis based on a mutant screen

**Fig. 2:**
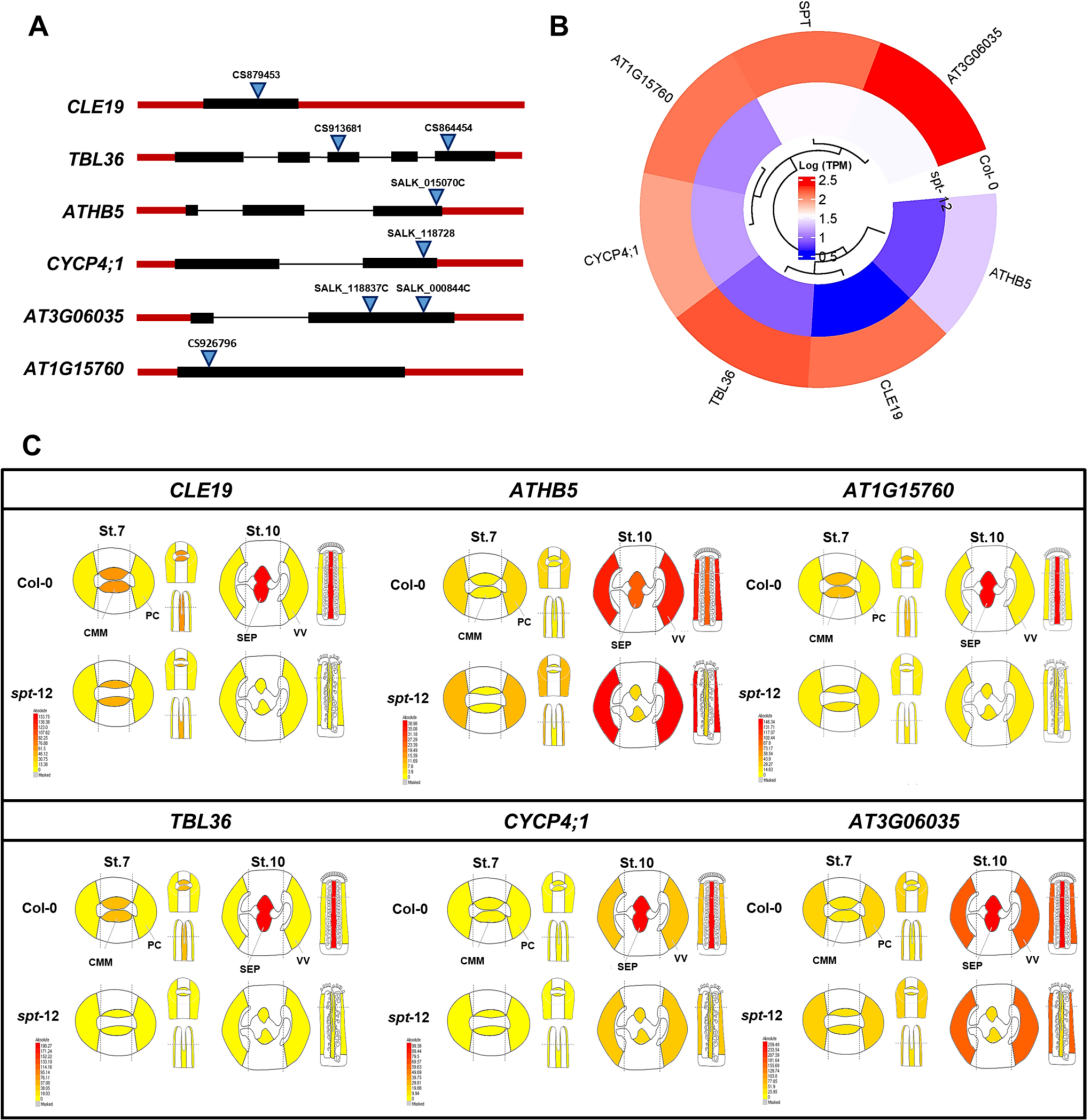
Comparison of transcriptional profiles of the 6 genes in wild-type (Col-0) and *spt-12* mutant gynoecia. **A** Schematic diagrams of gene structures and T-DNA insertion sites. The black boxes represent the exons, the red boxes represent the 5’-UTR and 3’-UTR regions, the black (thin) lines represent the introns, and the blue triangles represent the T-DNA insertion site. Gene orientation is from left to right. **B** Heatmap of transcriptional profile of genes in septum (Log_2_ TPM). **C** Tissue-specific expression patterns of the 6 genes in wild-type and *spt-12* mutant gynoecia (stages 7 and 10), available in the eFP browser (https://bar.utoronto.ca/efp_arabidopsis/cgi-bin/efpWeb.cgi?dataSource=Gynoecium). CMM = carpel margin meristem, PC = presumptive carpel wall, VV = valves, SEP = septum

Of the 25 genes evaluated using insertion lines [41T-DNA lines in total (for various genes more alleles were available), Fig. 1G, Table S3], we observed visible phenotypes in mutants for 6 genes (24%), namely for *CLE19*, *TBL36, ATHB5*, *CYCP4;1*, *AT3G06035*, and *AT1G15760* (Fig. 2A). For the first gene, *CLE19* is a gene belonging to the *CLAVATA3*/*EMBRYO SURROUNDING REGION* (*CLE*) family, which are a group of protein ligands involved in the regulation and differentiation of meristematic tissues in plants (Li et al. 2019), in addition to being involved in the regulation of seed development (Wang et al. 2016; Kucukoglu et al. 2020). The *TBL36* gene belongs to the Trichome Birefringence-LIKE (*TBL*) family containing the plant-specific DUF231 domain and is known to be involved in trichome initiation and the O-acetylation pathway of plant cell walls (Kabir et al. 2023; Dauphin et al. 2024). *ATHB5* belongs to the homeodomain leucine zipper (HD-Zip) family of transcription factors which is a plant-specific family. Some of these family members are involved in developmental processes including vascular tissue development, trichome development, meristem maintenance, and hormone action (Henriksson et al. 2005; Ariel et al. 2007). The *CYCP4;1* gene belongs to the P-type cyclins (*CYCPs*), which are a new class of cyclins in Arabidopsis (Chen et al. 2020; Li et al. 2024). *AtCYCPs* are enriched in proliferating cells and are moderately expressed in mature and differentiating tissues (Xu et al. 2020). Some genes that have been characterized are involved in cell cycle activation in the meristem (Peng et al. 2014). The *AT3G06035* gene belongs to the family of glycosylphosphatidylinositol-anchored proteins (GPI-AP). In Arabidopsis, it has been studied that GPI-APs participate in cell wall regulation processes (Zhou 2022). GPI-APs have also been reported to be important for root and shoot growth, fertility, stomata formation, and male gametophyte viability, by mediating directional growth of pollen tubes (Li et al. 2013; Bundy et al. 2016). Finally, the *AT1G15760* gene belongs to the group of proteins containing the SAM (sterile alpha motif) domain. Most of these proteins remain poorly characterized in plants, except for the transcription factor *LEAFY*, crucial for flower development (Sayou et al. 2016). In general, the SAM domain is known to play diverse and important roles in eukaryotes, acting as a protein–protein interaction domain and binding to a variety of substrates, including RNA and lipids (Denay et al. 2017; Olasz et al. 2024). Interestingly, most genes found here are not transcription factors, which normally attract attention in functional screens.

In Fig. 2B, the expression level of the 6 genes can be observed, in addition to *SPT*. In addition, in Fig. 2C, the expression profiles of the 6 genes are depicted based on the data availability in the eFP browser (https://bar.utoronto.ca/efp_arabidopsis/cgi-bin/efpWeb.cgi?dataSource=Gynoecium; Winter et al. 2007). By evaluating the expression level of these genes in specific tissues of the gynoecium (Fig. 2C): carpel wall (PC; stage 7), carpel margin meristem (CMM; stage 7), valves (VV; stage 10), and septum (SEP; stage 10), it can be observed that, in addition to all genes having lower expression in the septum in the *spt-12* mutant (stage 10), the genes *CLE19*, *TBL36,* and *AT1G15760* show a (slight) decreased expression level from stage 7 of the development of the gynoecium in the CMM. For the other genes, *ATHB5*, *CYCP4;1,* and *AT3G06035,* their expression level is low in the CMM both in Col-0 and in *spt-12*. In addition, *ATHB5*, *CYCP4;1,* and *AT3G06035* are also expressed in the valves in Col-0 and this expression is not altered in the *spt-12* mutant (Fig. 2C). The valves are a tissue where *SPT* is not expressed in the wild-type gynoecium (Heisler et al. 2001; Groszmann et al. 2010).

### Functional analysis of genes during flower and fruit development

The phenotypic analysis of the homozygous T-DNA mutant lines for the 6 genes: *CLE19*, *TBL36*, *ATHB5*, *CYCP4;1*, *AT3G06035,* and *AT1G15760* consisted of examining flowers, measuring the length of gynoecia, quantifying the number of ovules, measuring the length of fruits, and quantifying seeds and percentage of ovule abortion. First, homozygous lines were verified based on the presence of the T-DNA insertion by PCR (Fig. S1), subsequently, gene expression was analyzed to observe if the lines were knock out. The latter resulted in the observation of a strong reduction of gene expression in all lines, though in all lines still some minor expression was detected (Fig. S2), meaning they are not completely null lines, but it is to be expected that the gene function is lost or strongly reduced. Two alleles were available and analyzed for the genes *TBL36* and *AT3G06035*; similar phenotypes were observed and presented in Figs. S3–S7.

**Fig. 3:**
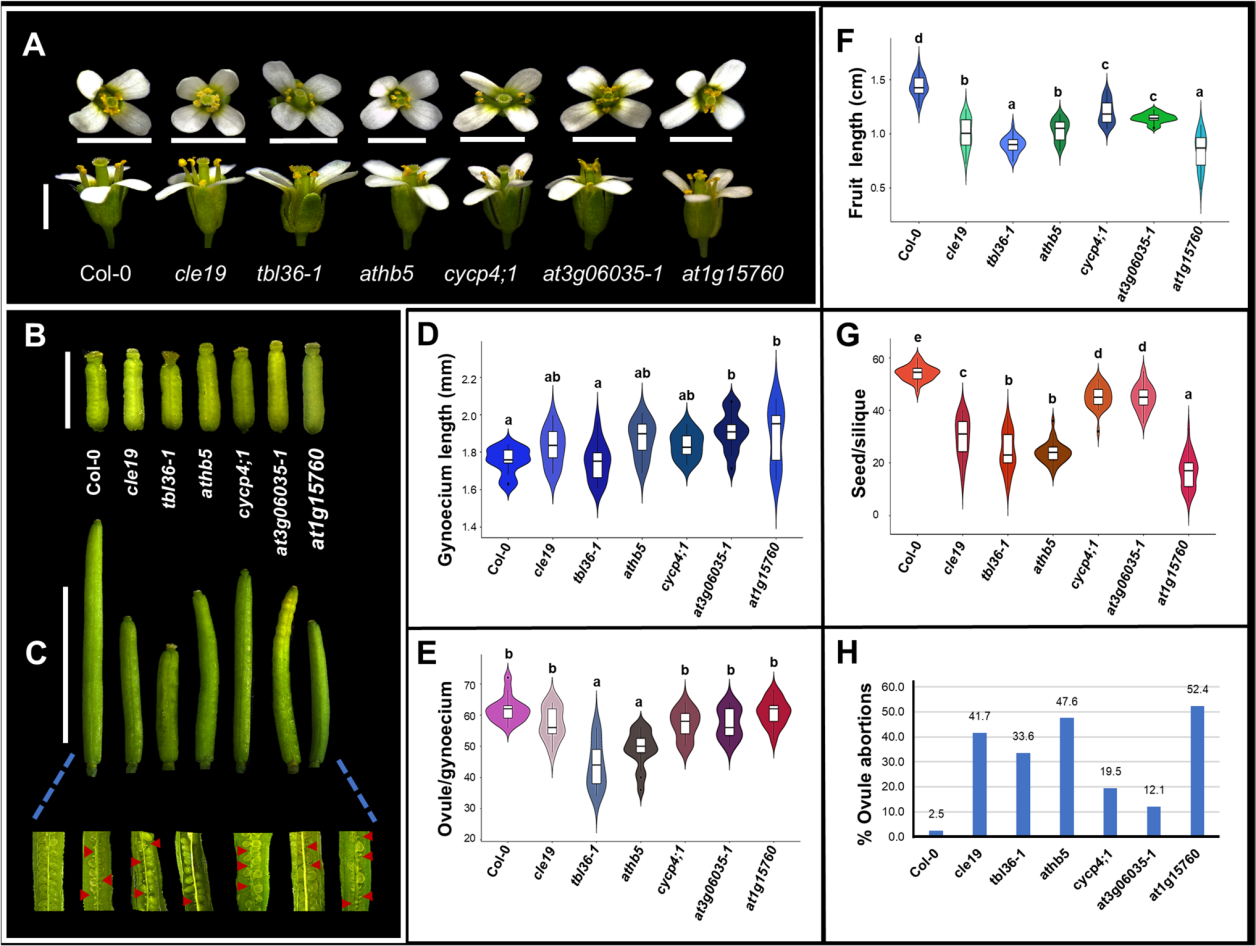
Phenotypic analysis of Arabidopsis wild‐type (Col-0) and mutant plants. **A** Representative phenotype of flowers at stage 13 (anthesis). **B** Mature gynoecia phenotypes (stage 12, according to Smyth et al. 1990). **C** Comparison of siliques from wild-type Col-0 and mutant plants. Red arrows indicate aborted ovules. **D**-**G** Violin plots of gynoecium length (*n* = 13), ovule number (*n* = 15), fruit length (*n* = 30), and seed number (*n* = 30). **H** Graphs with percentages of aborted ovules (*n* = 20). Different letters indicate a statistically significant difference, based on an ANOVA followed by Tukey´s honest significance (Tukey HSD) test, *P* < 0.05. Scale bars = 2 mm (**A**, **B**), 1 cm (**C**)

First, flower development was analyzed in the mutants. In general, the flowers showed no obvious visible alterations (Figs. 3A and S3A). Regarding the length of the gynoecium, according to the statistical analysis (*n* = 13, *P* < 0.05), a statistical difference was found for the mutants *at3g06035-1* and *at1g15760*, their gynoecia are longer than in wild-type Col-0 plants. In the other mutants, no significant statistical difference was found, although the mutants *cle19*, *athb5* and *cycp4;1* presented slightly longer gynoecia than Col-0 (Figs. 3B, D and S3B, D). Regarding the number of ovules, the mutants *tbl36-1* and *athb5* (*n* = 15, *P* < 0.05) presented a lower number of ovules compared to wild type (Col-0). The other mutants did not show a significant statistical difference in the production of ovules with respect to wild-type plants (Figs. 3E and S3E).

On the other hand, when examining the fruits, all mutants presented fruits with the presence of aborted ovules (Figs. 3C and S3C). When measuring the length of the fruit and counting the number of seeds, a significant statistical difference was found (*n* = 30, *P* < 0.05), all mutant plants presented a shorter fruit length and lower seed production compared to wild-type plants. The *tbl36-1* and *at1g15760* mutants presented the shortest fruits, and the *at1g15760* mutant showed the lowest number of seeds (Figs. 3C, F, G, S3C, S4A, B). Regarding the percentage of aborted ovules, the *at1g15760* mutant presented the highest percentage with 52.4%, followed by *athb5* (47.6%), *cle19* (41.7%), and *tbl36-1* (33.6%) (Figs. 3H and S4C).

**Fig. 4:**
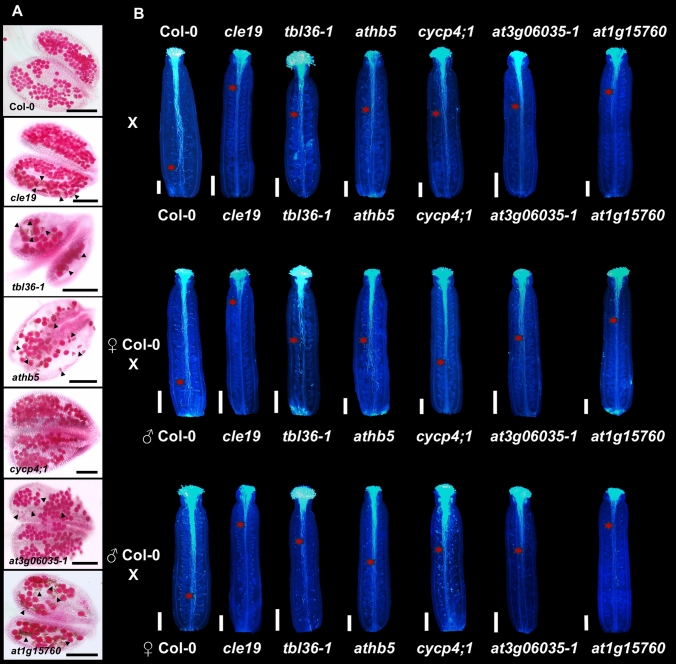
Staining of pollen grains and pollen tubes in wild-type (Col-0) and mutant plants. **A** Pollen viability analysis by Peterson’s staining. Arrowheads indicate aborted pollen grains. **B** Gynoecia from self-pollinated and reciprocal crosses with pollen tubes stained with aniline blue. Red asterisks indicate the growth front of pollen tubes. Scale bars = 100 µm (**A**), 200 µm (**B**)

**Fig. 5:**
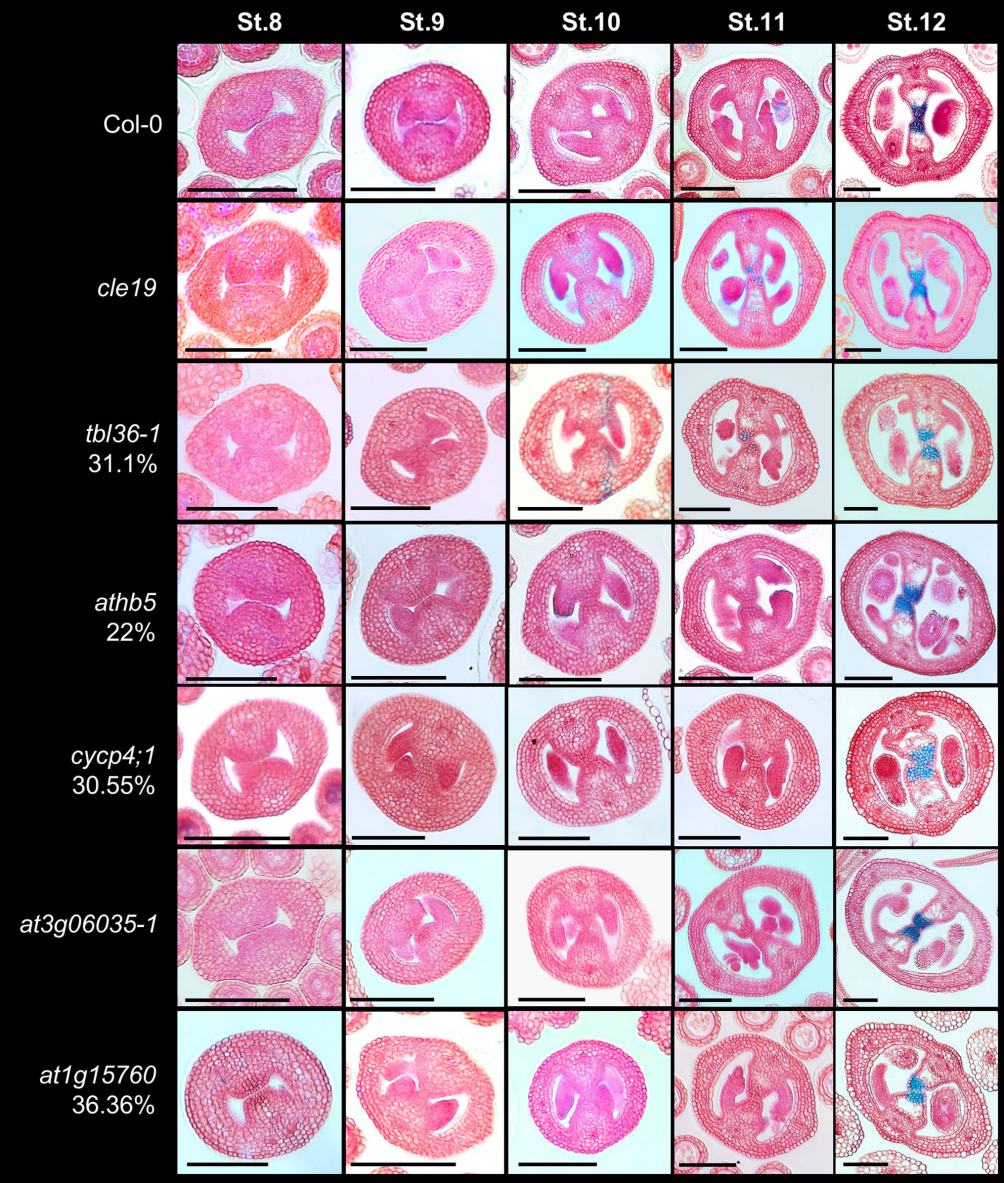
Transverse gynoecia sections at different stages (stages 8–12, according to Smyth et al. 1990) in wild-type (Col-0) and mutant plants. Blue staining indicates the transmitting tract. Scale bars = 100 µm. St = Stage of development of the gynoecium. Col-0, *n* = 66; *cle19, n* = 55; *tbl36-1, n* = 64; *athb5, n* = 59; *cycp4;1, n* = 51; *at3g06035-1*, *n* = 67; *at1g15760, n* = 66

To explain the decrease in the number of seeds, the role of the genes was further investigated to see if the condition is due to the male or female defect. Pollen viability was analyzed using Peterson staining, which allows the viability of pollen grains to be seen (Peterson et al. 2010). As a result, it was observed that all mutants, except *cycp4;1*, presented some aborted pollen grains; however, large quantities of viable pollen grains were observed (Figs. 5A and S5A). Subsequently, we analyzed pollen tube growth, that has to pass through the medial tissues inside the gynoecium. For this, we emasculated flowers and hand pollinated; 24 h after, the gynoecia were fixed and stained with aniline blue to observe pollen tubes. In all mutants, following manual self-pollination, a clear reduction in pollen tube growth was observed compared to wild type; pollen tube growth was observed mostly in the apical part or up to half of the ovary (Fig. 4B). In the case of reciprocal crosses, when wild-type gynoecia were pollinated with mutant pollen, the defect in the pollen was confirmed (Figs. 4B and S5B). Similarly, when pollinating mutant gynoecia with pollen from wild type, the growth of the pollen tubes remained mostly in the apical and middle part of the gynoecium, suggesting also a defect on the female side, the gynoecium (Figs. 4B and S5B).

After observing pollen and pollen tube growth, we examined the development of the internal tissues of the gynoecium of the mutants. We made thin transverse gynoecia sections and stained them with alcian blue and neutral red, allowing to also see the transmitting tract (blue color). We observed in most mutants normal development of the gynoecium. However, in the *tbl36-1* (31.1%, *n* = 64), *athb5* (22%, *n* = 59), *cycp4;1* (30.55%, *n* = 51), and *at1g15760* (36.36%, *n* = 66) mutants, a percentage of gynoecia presented a defect in the fusion of the septum. In addition, in the *tbl36-1* mutant, a reduced presence of the transmitting tract was observed, and in the *cycp4;1* mutant the septum and replum were observed to be wider due to an increased cell number compared to wild type (Figs. 5, S6 and S7).

## Discussion

The aim of this study was to identify new regulators of gynoecium development. For this, we used transcriptome data from Laser-Assisted Microdissection (LAM) combined with RNA-seq from wild type and the *spt-12* mutant, a mutant affected in medial tissue development (Luna-García et al. 2024; Luna-García et al., data not shown). In the present work we found that of the 25 genes selected for functional analysis, mutants for 6 genes showed reduced fertility (*CLE19*, *TBL36*, *ATHB5*, *CYCP4;1*, *AT3G06035* and *AT1G15760*), mainly a lower number of seeds and shorter fruit length, and the presence of ovule abortion was observed (Table 1). Despite the *ATHB5* gene, the other five genes functionally analyzed, are not transcription factors, which shows that in addition to transcription factors, other proteins are also important for the development of the gynoecium. In addition, for most of these genes there are a few or no reports on their possible functionality in the development of the flower (gynoecium) or fruit. With the exception of the *CLE19* gene, previous works reported that this peptide affects fertility in Arabidopsis. Xu et al. (2015) demonstrated that *CLE19* is expressed in the embryo and that it is involved in the development of the embryo and endosperm. The phenotype of the mutant presented seed abortion, with defective establishment of cotyledons (Xu et al. 2015). Later, Wang et al. (2017) observed defects in the male reproductive organ in Arabidopsis, finding that this peptide is necessary for normal pollen development, pollen germination, and pollen tube elongation. The mutant presented defects in the formation of the pollen exine; in addition, they reported that this gene controls the synthesis of lipid, phenolic, and flavonoid components necessary for the formation of the pollen wall (Wang et al. 2017). Our results confirmed the previous studies; we also observed defects in growth of pollen tubes, since growth was observed mostly in the apical part of the gynoecium, unlike in wild-type gynoecia. In addition, we observed ovule abortion (41.7%) in the *cle19* mutant.

**Table 1:** Phenotypic summary of mutant lines compared to wild-type plants

Phenotype	*cle19*	*tbl36-1*	*athb5*	*cycp4;1*	*at3g06035-1*	*at1g15760*
Gynoecium length	=	=	=	=	↑	↑
Number of ovules	=	↓	↓	=	=	=
Number of seeds	↓	↓	↓	↓	↓	↓
Fruit length	↓	↓	↓	↓	↓	↓
Aborted ovules	✓	✓	✓	✓	✓	✓
Aborted pollen grains	✓	✓	✓	✗	✓	✓
Pollen tube growth defect	✓	✓	✓	✓	✓	✓
Septum fusion defect	✗	✓	✓	✓	✗	✓
Transmitting tract defect	✗	✓	✗	✗	✗	✗

Symbol key:

**= **No difference compared to Col-0 (wild-type)

↑ Increase compared to Col-0 (e.g., longer gynoecium length)

↓ Decrease compared to Col-0 (e.g., fewer ovules, seeds, or shorter fruit length)

✓ Presence of the phenotype or developmental defect

✗ Absence of the phenotype; normal development

On the other hand, when measuring the length of gynoecia, we observed a slight increase in the *at3g06035-1* and *at1g15760* mutants compared to wild type. Furthermore, we observed a statistically significant reduction in the number of ovules in the *tbl36-1* and *athb5* mutants, although ovule production was slightly reduced in most of the mutants. The number of ovules has been shown to be determined at early stages of floral development, where the establishment and maintenance of the meristematic activity of the CMM is inherently correlated with the generation of ovule primordia, in addition to the fact that ovule number is an interconnected trait that affects seed yield (Cucinotta et al. 2014, 2020; Qadir et al. 2021). In the *spt* mutant, a reduced number of cells in the CMM is present, and consequently, a reduction in ovule production is observed, resulting in reduced seed number and fruit length (Alvarez and Smyth 1999; Nahar et al. 2012; Reyes-Olalde et al. 2017).

Furthermore, according to our results, we observed defects in both the male and female organs. In the male organ (the anthers), we observed the presence of some aborted pollen grains in all the mutants except *cypc4;1*, and we observed a defect in the growth of pollen tubes in all the mutants. Pollen tube growth was observed only in the apical or middle part of the gynoecium, unlike in the wild-type gynoecium. In addition, when pollinating mutant gynoecia with wild-type pollen, the same phenotype was observed, which indicates that there is also a defect on the female side (gynoecium). One of the important aspects during pollen development is the formation of the pollen wall since, in addition to protecting the male gametes, preventing pollen degradation and promoting its germination, it is also important for the polar growth of the pollen tube (Ma et al. 2021; Wei and Ma 2023). During the reproductive process, the pollen tube must pass through different tissues of the gynoecium: stigma, style, and transmitting tract, before it can reach the ovules (Mizuta and Higashiyama 2018; Pereira et al. 2021). This is achieved by polar or tip growth of the pollen tube, which is a self-organizing process that requires a close coordination of a multitude of processes, such as F-actin cytoskeletal dynamics, vesicle trafficking, ion fluxes, signaling events such as Ca^2+^ and GTPase mediated signaling, exocytosis and endocytosis, as well as cell wall modification and reorganization (Guan et al. 2013; Guo and Yang 2020).

Analyzing the female side further, cross sections of the gynoecia stained with alcian blue and neutral red allowed us to observe that in all mutants, normal development of the gynoecium tissues was observed, with normal development of the septum and the presence of the transmitting tract. However, in the *tbl36-1*, *athb5*, *cycp4;1,* and *at1g15760* mutants, a small percentage showed an effect in the fusion and formation of the septum. Furthermore, in the *tbl36-1* mutant a decrease of transmitting tract differentiation was observed, since we observed unstained cells in the center of the septum, and in the *cycp4;1* mutant the septum and replum appeared to be wider than wild type. Postgenital organ fusion is known to be a complex process that depends on surface contact, involving contact-mediated cell adhesion as well as changes in the epidermal cells in contact (Lolle and Pruitt 1999; Zhong and Preston 2015). Furthermore, cell wall composition and modifications have been reported to be important for postgenital carpel fusion in Arabidopsis and to contribute to proper septum development and transmitting tract formation (Herrera-Ubaldo and de Folter 2018). In Arabidopsis, the transmitting tract is formed through a programmed cell death process, which provides sufficient intercellular space to allow pollen tube growth and has been proposed to have multiple functions in pollen tube guidance, nutrition, defense, and adhesion (Cascallares et al. 2020; Pereira et al. 2021). The transmitting tract cells produce an extracellular matrix (ECM) containing a mixture of glycoproteins, glycolipids, and polysaccharides (Lennon et al. 1998; Crawford and Yanofsky 2008, 2011). Therefore, when the septum and/or the transmitting tract development are affected, as well as alterations in the production of the extracellular matrix, plant fertility is reduced. Examples of genes involved in the development of the septum and/or transmitting tract, in addition to *SPT*, are *NTT* (Crawford et al. 2007), *HEC* (*HEC1, 2, 3*) (Gremski et al. 2007), *HAF*, *BEE1* and *BEE3* (Crawford and Yanofsky 2011), *STK* (Herrera-Ubaldo et al. 2019), and *TET1*/*TRN2* (Luna-García et al. 2024). In mutants for the mentioned genes, pollen tube growth is drastically affected.

The correct development of the gynoecium pattern is also determined by genes involved in the cell cycle. As mentioned above, the CMM tissue contains cells with meristematic activity and subsequently gives rise to the tissues of the medial domain, including the septum. According to transcriptomic data, it is known that genes related to the cell cycle, such as CDKs and CYCs, are expressed in the CMM and septum (Luna-García et al. 2024). In our results, the *cycp4;1* mutant, which is a cyclin, presented altered development of the septum and replum; in these tissues a greater number of cells were observed compared to in wild-type gynoecia. *CYCP4;1* belongs to the P-type cyclins (CYCP), which are a new class of cyclins in Arabidopsis that have been little studied (Chen et al. 2020; Li et al. 2024). Recently, it has been reported that SPT negatively controls cell proliferation and directly represses the expression of *CYCP3;1* and *CYCP3;2* in the style, in opposition to the cell proliferation signal provided by cytokinins. Furthermore, the authors demonstrated that *CYCP3s* activity is responsible for the unfused style phenotype of the *spt* mutant, concluding that these cyclins are involved in the control of the establishment of radial symmetry of the style (Jamil et al. 2024). Interestingly, in the transcriptomic data *CYCP3;1* is one of the genes that are expressed in the septum tissue of the *spt* mutant, like *CYCP4;1* although with an opposite expression level. Moreover, other recent reports also demonstrated that SPT regulates cyclins such as D1 type and D3 type (Cerbantez-Bueno et al. 2024; Tasker‐Brown et al. 2024). These results make it interesting to investigate further the functionality of cyclins during flower and gynoecium development.

As mentioned above, pollen development, pollen tube growth, septum formation and fusion, and transmitting tract differentiation are accompanied by processes involving cell wall modifications. Interestingly, the genes *TBL36* and *AT3G06035* belong to families that are generally assigned to be involved in processes influencing cell wall modification. The first gene is involved in the cell wall O-acetylation pathway (Kabir et al. 2023; Dauphin et al. 2024), and the second gene encodes a glycosylphosphatidylinositol-anchored protein (GPI-AP), a class of protein that has been reported to be involved in cell wall regulation processes (Zhou 2022). GPI-AP proteins have been reported to be involved in the maintenance and integrity of the Arabidopsis pollen tube, whose mutations cause male sterility, with low pollen germination rate, reduced pollen tube growth, and seed abortion in Arabidopsis such as *COBL10* (Li et al. 2013), *COBL11* (Li et al. 2023), *AGP6* and *AGP11* (Levitin et al. 2008; Coimbra et al. 2010), *FLA3* (Li et al. 2010), and *A36* and *A39* (Gao et al. 2017).

## Conclusion

In this work we found that the genes *CLE19*, *TBL36*, *ATHB5*, *CYCP4;1*, *AT3G06035,* and *AT1G15760* are involved in flower development, both male and female organs were affected in mutants, resulting in reduced seed production and fruit length. With this we contribute to the understanding of the genetic regulatory network that orchestrates the development of the gynoecium and fruit. In future work, it will be interesting to evaluate double mutants with the SPT transcription factor, to better understand how these genes contribute to the development of reproductive tissues and organs in plants.

## Supplementary Information

Below is the link to the electronic supplementary material.

Supplementary file1 Fig. S1 Identification of T-DNA insertion lines by PCR for *CLE19*, *ATHB5*, *TBL36*, *CYCP4;1*, *AT1G15760*, and *AT3G06035*. Fig. S2 Semi-quantitative RT-PCR analysis of gene expression in inflorescences of Arabidopsis mutants and wild-type (Col-0). Fig. S3 Phenotypic analysis of Arabidopsis wild‐type (Col-0), *tbl36-2*, and *at3g06035-2* mutant plants. Fig. S4 Statistical analysis on fruits and seeds from Arabidopsis wild-type (Col-0), *tbl36-2*, and *at3g06035-2* mutant plants. Fig. S5 Staining of pollen grains and pollen tubes in Arabidopsis wild-type (Col-0), *tbl36-2*, and *at3g06035-2* mutant plants. Fig. S6 Transverse gynoecia sections at different developmental stages (stage 8–12) in Arabidopsis wild-type (Col-0), *tbl36-2*, and *at3g06035-2* mutant plants. Fig. S7 Quantification of cell number in the septum and replum of the *cycp4;1* mutant, and wild-type (Col-0). Table S4 Primer sequences used in this study (PDF 1679 KB)

Supplementary file2 Table S1 Differentially expressed genes (SEPcol vs SEPspt-12), FDR ≤ 0.05, Log2FC ≥ 1. Table S2 GO enrichment analysis data for down-regulated spt genes. Table S3 Genes and T-DNA lines selected for this study (XLSX 194 KB)

## Data Availability

The datasets generated during and/or analyzed during the current study are available from the corresponding author upon reasonable request. The RNA-seq data are available in the European Nucleotide Archive (ENA) under accession numbers PRJEB65130 (Col-0) and PRJEB79454 (*spt-12*), and in the eFP browser (Winter et al., 2007): https://bar.utoronto.ca/efp_arabidopsis/cgi-bin/efpWeb.cgi?dataSource=Gynoecium.

## Abbreviations

CMM: Carpel margin meristem
Col-0: Arabidopsis Columbia-0
DEG: Differentially expressed gene
PC: Presumptive carpel wall
SEP: Septum
SPT: SPATULA
TPM: Transcripts per million
