# Supplementary material for: Identifying new players of gynoecium development using tissue-specific transcriptome data of Arabidopsis
Source: Planta. 2025 Jul 31;262(3):67. doi: 10.1007/s00425-025-04784-0 (PMC12313761; doi:10.1007/s00425-025-04784-0)
Supplement: Supplementary file 1 — Supplementary file1 Fig. S1 Identification of T-DNA insertion lines by PCR for CLE19, ATHB5, TBL36, CYCP4;1, AT1G15760, and AT3G06035. Fig. S2 Semi-quantitative RT-PCR analysis of gene expression in inflorescences of Arabidopsis mutants and wild-type (Col-0). Fig. S3 Phenotypic analysis of Arabidopsis wild‐type (Col-0), tbl36-2, and at3g06035-2 mutant plants. Fig. S4 Statistical analysis on fruits and seeds from Arabidopsis wild-type (Col-0), tbl36-2, and at3g06035-2 mutant plants. Fig. S5 Staining of pollen grains and pollen tubes in Arabidopsis wild-type (Col-0), tbl36-2, and at3g06035-2 mutant plants. Fig. S6 Transverse gynoecia sections at different developmental stages (stage 8–12) in Arabidopsis wild-type (Col-0), tbl36-2, and at3g06035-2 mutant plants. Fig. S7 Quantification of cell number in the septum and replum of the cycp4;1 mutant, and wild-type (Col-0). Table S4 Primer sequences used in this study (PDF 1679 KB) [file 425_2025_4784_MOESM1_ESM.pdf]

Identifying new players of gynoecium development using tissue-specific transcriptome data of Arabidopsis

Eliana Arias-Pérez[1], Valentín Luna-Garcia[1], Judith J. Bernal-Gallardo[1], and Stefan de Folter[1*]

[1] Unidad de Genómica Avanzada (UGA-Langebio), Centro de Investigación y de Estudios Avanzados del Instituto Politécnico Nacional (Cinvestav), 36824 Irapuato, México.

[*] Corresponding author: Stefan de Folter, stefan.defolter@cinvestav.mx

ORCID ID: 0000-0003-4363-7274

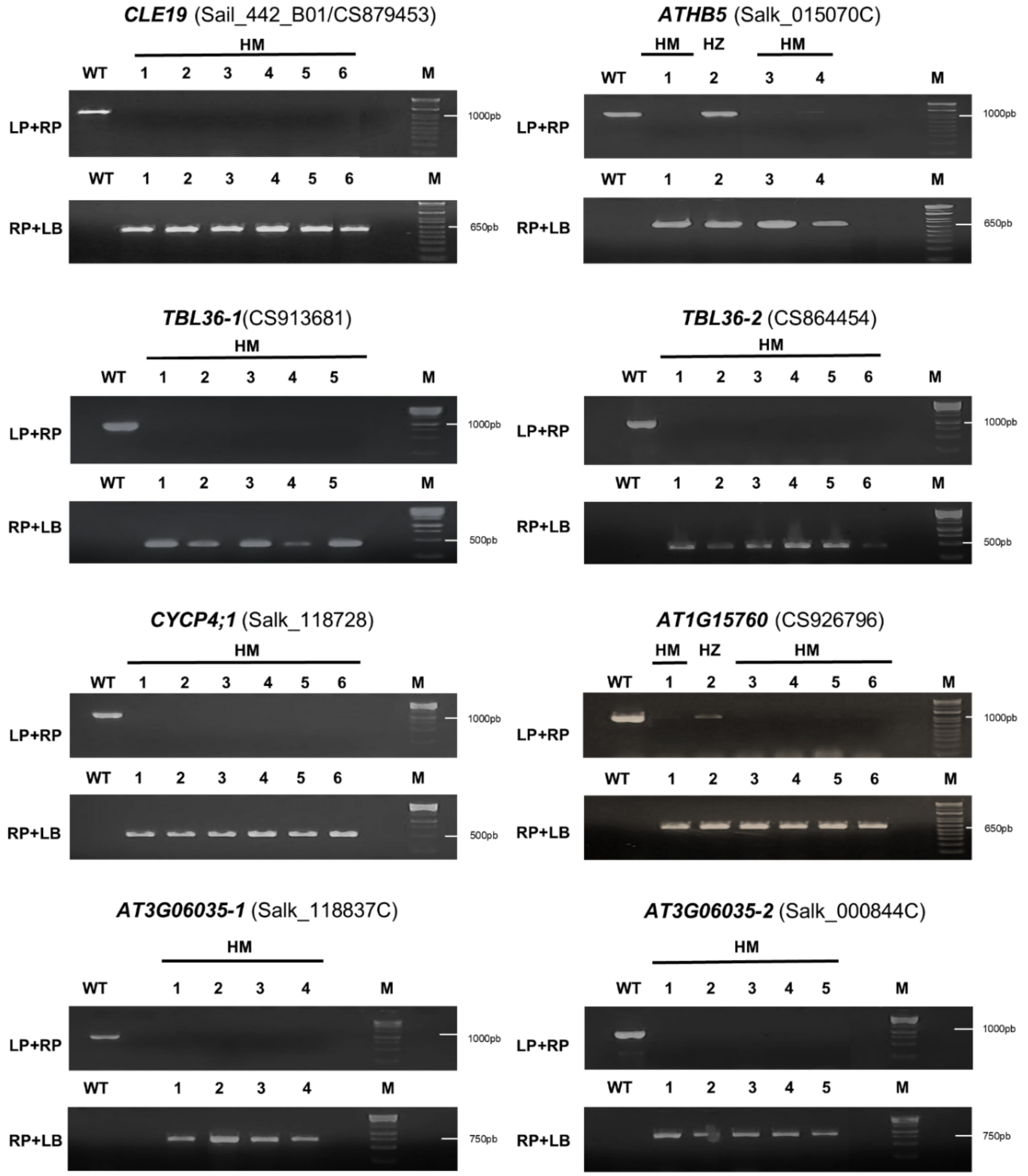

Fig. S1 Identification of T-DNA insertion lines by PCR for *CLE19*, *ATHB5*, *TBL36, CYCP4;1*, *AT1G15760*, and *AT3G06035*. PCR products loaded on gels; fragment size indicated. LP: Left primer; RP: Right primer and LB: Left border primer, HZ: Heterozygous, HM: Homozygous. M: 1 kb marker (1kb Plus DNA Ladder [in gels with the product of CLE19, ATHB5, AT1G15760] and GeneRuler 1kb DNA Ladder [in the rest of the gels], Thermo Fisher Scientific).

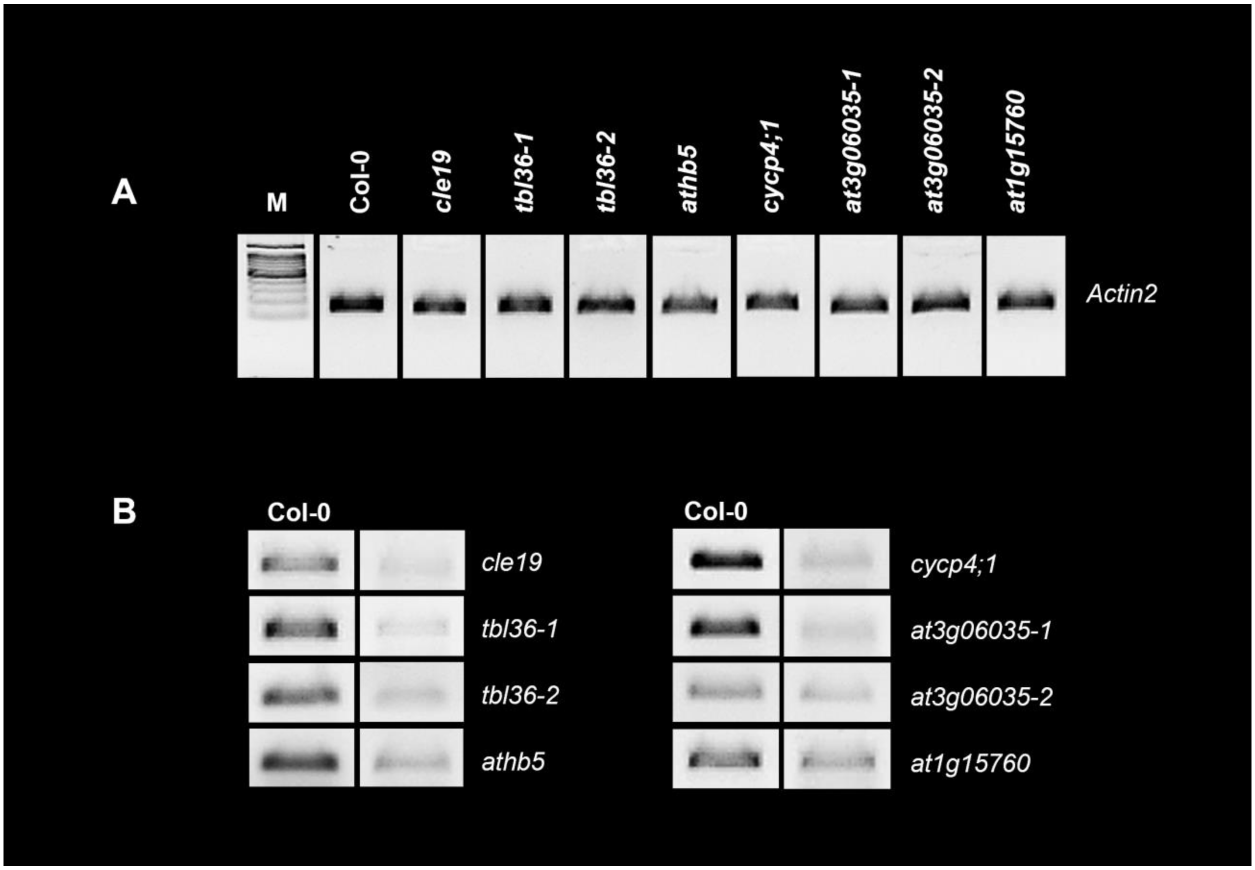

Fig. S2 Semi-quantitative RT-PCR analysis of gene expression in inflorescences of the mutants and wild-type (Col-0). **A**. PCR products loaded on gels. *Actin2* was used as an internal control gene. **B**. PCR products loaded on gels. Gene expression of the different genes of interest in the corresponding mutant lines and wild-type (Col-0). M: 1 kb marker (GeneRuler 1kb DNA Ladder, Thermo Fisher Scientific)

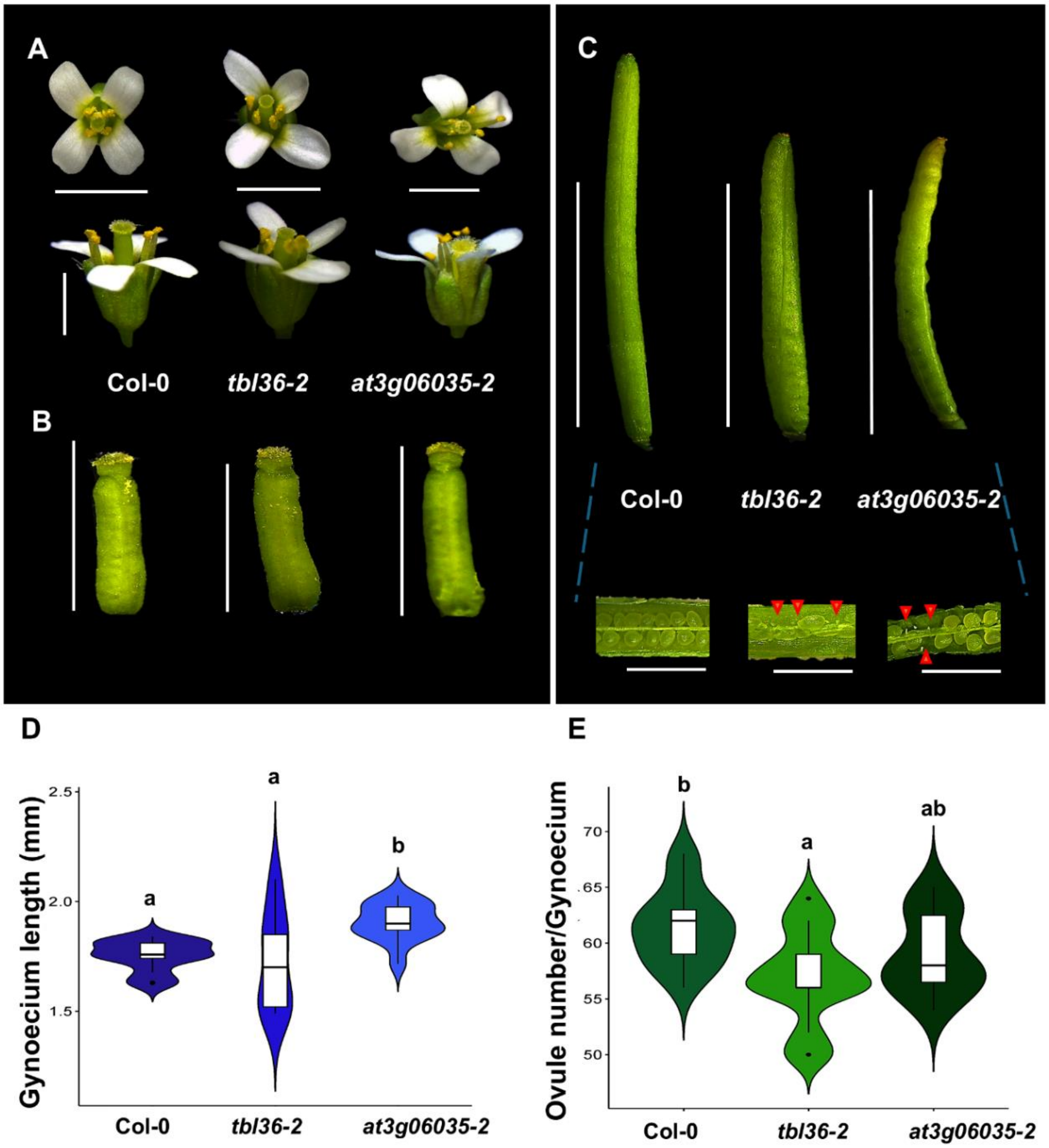

Fig. S3 Phenotypic analysis of Arabidopsis wild-type (Col-0), *tbl36-2*, and *at3g06035-2* mutant plants. **A**. Representative phenotype of flowers at stage 13 (anthesis). **B**. Mature gynoecia phenotypes (stage 12, according to Smyth et al., 1990). **C**. Comparison of siliques from Col-0 and mutant plants. Red arrows indicate aborted ovules. **D**-**E**. Statistical analyses of gynoecium length and ovule number. Different letters indicate a statistically significant difference, based on an ANOVA followed by Tukey´s honest significance (Tukey HSD) test, gynoecium length n = 13, Ovule number/gynoecium n=15, P <0.05. Scale bars = 2 mm (**A**, **B**), 1 cm (C).

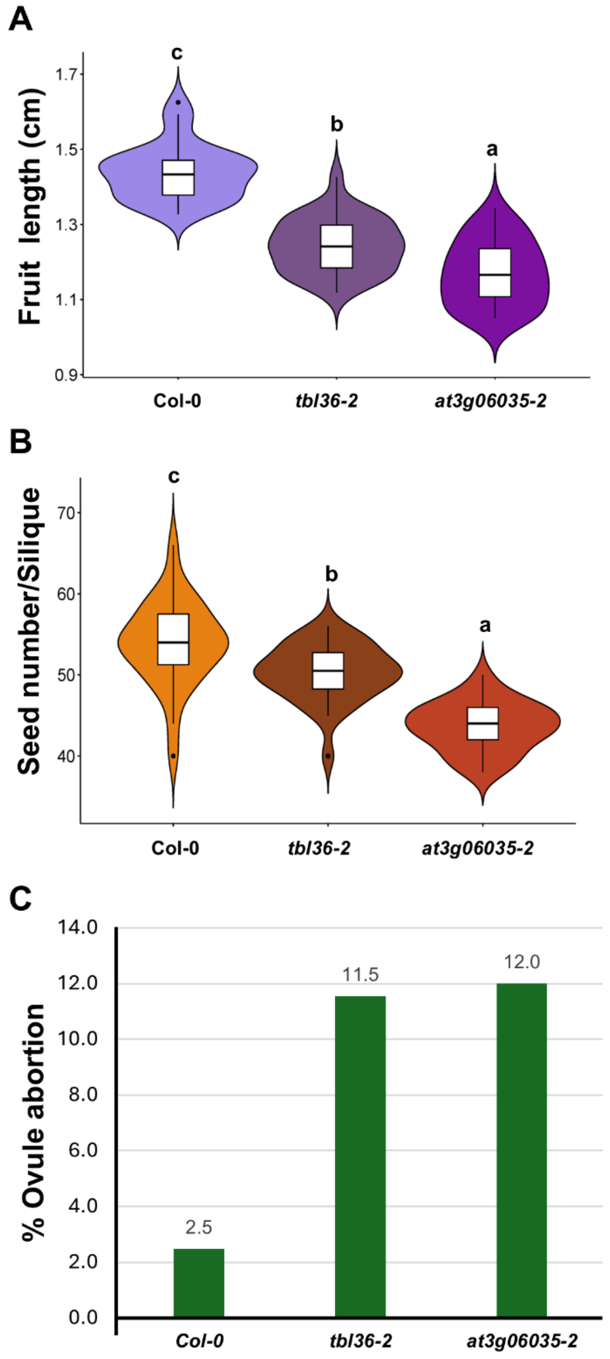

Fig. S4 Statistical analysis on fruits and seeds from wild-type (Col-0), *tbl36-2*, and *at3g06035-2* mutant plants. **A**-**B**. Fruit length and number seed, respectively. Different letters above the plots indicate a statistically significant difference, based on an ANOVA and Tukey as *post hoc* (Tukey HSD) test, n = 30, P <0.05. **C**. Percentage of aborted ovules (n = 20).

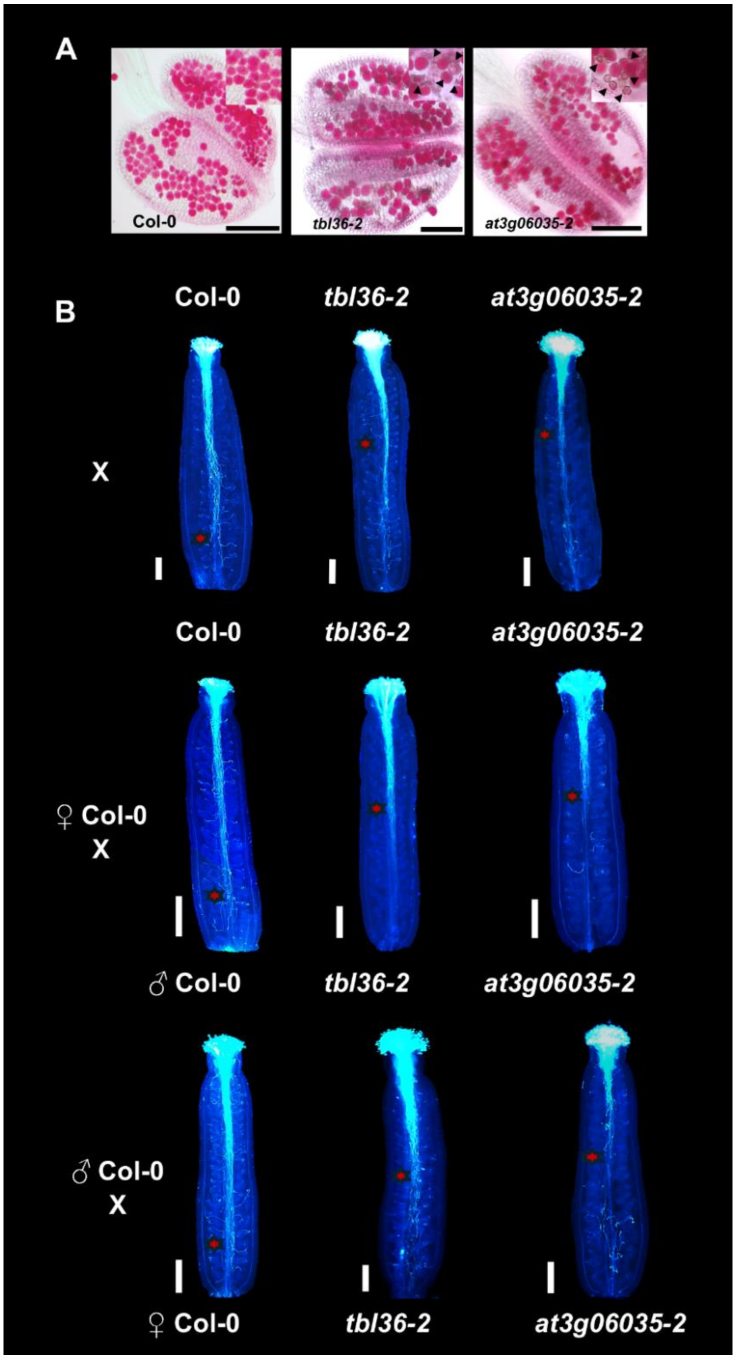

Fig. S5 Staining of pollen grains and pollen tubes in wild-type (Col-0), *tbl36-2,* and *at3g06035-2* mutant plants. **A**. Pollen viability analysis by Peterson's staining. Arrowheads indicate aborted pollen grains. **B**. Gynoecia from self-pollinated and reciprocal crosses between the mutant plants and the wild-type Col-0, pollen tube growth was visualized using aniline blue staining. Red asterisks indicate the growth front of pollen tubes. Scale bars = 100 μm (**A**), 200 μm (**B**).

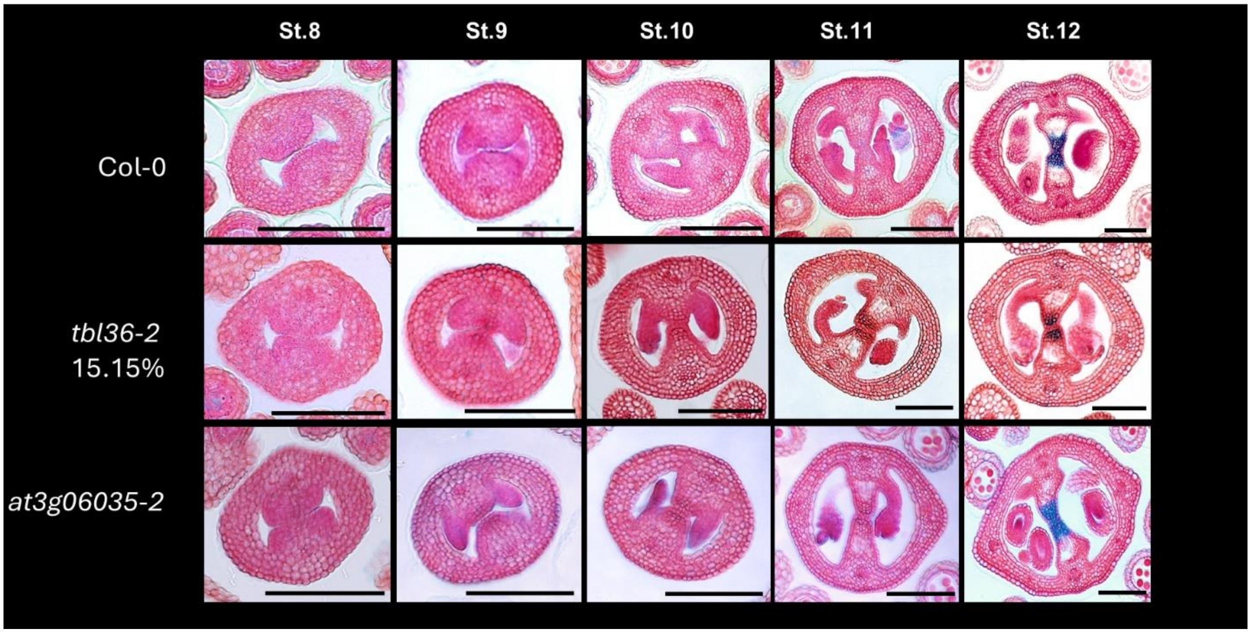

Fig. S6 Transverse gynoecia sections at different developmental stages (stage 8-12) in wild-type (Col-0), *tbl36-2*, and *at3g06035-2* mutant plants. In *tbl36-2*, 15.15% of the gynoecia showed defects in transmitting tract development, with unstained cells observed in the center of the septum. St= Developmental stage of the gynoecium; stages according to Smyth et al., (1990); *tbl36-2* (*n* = 55), *at3g06035* (*n* = 51). Scale bars = 100 µm.

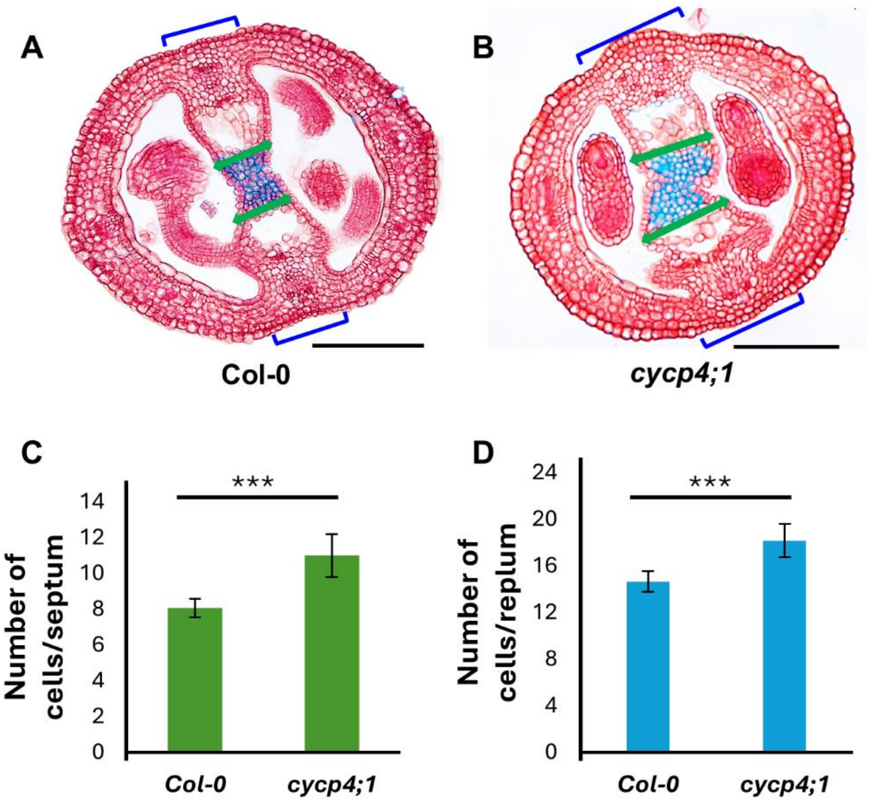

Fig. S7 Quantification of cell number in the septum and replum of the *cycp4;1* mutant and wild-type (Col-0). **A**-**B**. Cross sections of gynoecia of the *cycp4;1* mutant and wild-type (Col-0). Blue brackets indicate the cell counting area for the replum. Double-headed green arrow indicates the counting area for the septum (width). **C**-**D**. Student's t-test was used to evaluate significant differences in cell number for septum width (**C**) and replum (**D**) between Col-0 and *cycp4;1*. n = 12, asterisks represent statistical significance (*** P <0.001). Scale bars = 100 μm (**A**, **B**).

Table S4. Primer sequences used in this study.

Gene	Forward primer	Reverse primer	Product length LB+RP (bp)
CLE19	TGGGAGTTGGGAGACACTATG	GGCCACTTAATGAGGCTAGG	561-861
TBL36-1	ATCTCATTCCAAATATCCGGG	TAGTGGCTCTTTCCATGATGG	456-756
TBL36-2	ACTTAATCACCGCAATGTTCG	TGGGATTTGTGACAGTAACGC	462-762
ATHB5	GAAGACAAAGCAGCTTGAACG	TGAGTAATGCATTTTCCGACC	562-862
CYCP4;1	TGAGTCGACCAACGATAACG	TAGCCAGCCAGCCTGTAAAG	429-729
AT3G06035-1	TCCGAGAATATCCTGCATTTG	TTCAGGATCTTTAGATCTGGTCTG	590-890
AT3G06035-2	AGAGCCCAATTCGAGAGCTAC	CTAGCTGACGAAATCGCAGAC	434-734
AT1G15760	TGGTGGAAGAACCCTAAAACC	AATGTAATCCCCATTTCCTCG	452-752

Primer sequences used for semi-quantitative RT-PCR:

Gene	Forward primer	Reverse primer	Product length (bp)
CLE19	CTCCATGGGAACGATCGC	GTCCTTACAACACACAAGAAC	142
TBL36-1	GCTCCTCTGCTTGTGGAACT	CACCAGTGAGCGGAATCGAA	128
TBL36-2	ACGGTCATCCTTCGGTGTTC	GCAAGCACCAATGACTGCAA	96
ATHB5	CCTCTTTTATTGTTCTCGTCGTCC	TTAGCATCGATCGTATCATGTCG	168
CYCP4;1	TACAGGCTGGCTGGCTAAAG	CATGTTTCTGATCTGACATGGGT	153
AT3G06035-1	GACGCTTATGGCATGTGATGT	TCCAGATTTGTGTCGGTCGT	109
AT3G06035-2	TGTCGGTGAGTTTGGTGGAG	TGGGCCTTCTTATTGGGCTT	120
AT1G15760	CCAGAGTCGTGACTGCGATT	TCCACTTCCCAATCCCGTTG	144
ACTIN2	AATCACAGCACTTGCACC	ATTCCTGGACCTGCCTC	159

Sequence (LB):

LBb1.3	TTTTGCCGATTTCGGAAC
L4	TGATCCATGTAGATTTCCCGGACATGAAG
p745	AACGTCCGCAATGTGTTATTAAGTTGTC
LB3	TAGCATCTGAATTTCATAACCAATCTCGATACAC
